# Visual attractiveness is leaky: the asymmetrical relationship between face and hair

**DOI:** 10.3389/fpsyg.2015.00377

**Published:** 2015-04-09

**Authors:** Chihiro Saegusa, Janis Intoy, Shinsuke Shimojo

**Affiliations:** ^1^R & D-Kansei Science Research, Kao CorporationTokyo, Japan; ^2^Division of Biology and Biological Engineering, California Institute of TechnologyPasadena, CA, USA; ^3^Research Center for Advanced Science and Technology, The University of TokyoTokyo, Japan; ^4^Division of Engineering and Applied Sciences, California Institute of TechnologyPasadena, CA, USA; ^5^Computation and Neural Systems, California Institute of TechnologyPasadena, CA, USA

**Keywords:** attractiveness, face perception, emotion, information integrality, eye movement

## Abstract

Predicting personality is crucial when communicating with people. It has been revealed that the perceived attractiveness or beauty of the face is a cue. As shown in the well-known “what is beautiful is good” stereotype, perceived attractiveness is often associated with desirable personality. Although such research on attractiveness used mainly the face isolated from other body parts, the face is not always seen in isolation in the real world. Rather, it is surrounded by one’s hairstyle, and is perceived as a part of total presence. In human vision, perceptual organization/integration occurs mostly in a bottom up, task-irrelevant fashion. This raises an intriguing possibility that task-irrelevant stimulus that is perceptually integrated with a target may influence our affective evaluation. In such a case, there should be a mutual influence between attractiveness perception of the face and surrounding hair, since they are assumed to share strong and unique perceptual organization. In the current study, we examined the influence of a task-irrelevant stimulus on our attractiveness evaluation, using face and hair as stimuli. The results revealed asymmetrical influences in the evaluation of one while ignoring the other. When hair was task-irrelevant, it still affected attractiveness of the face, but only if the hair itself had never been evaluated by the same evaluator. On the other hand, the face affected the hair regardless of whether the face itself was evaluated before. This has intriguing implications on the asymmetry between face and hair, and perceptual integration between them in general. Together with data from a *post hoc* questionnaire, it is suggested that both implicit non-selective and explicit selective processes contribute to attractiveness evaluation. The findings provide an understanding of attractiveness perception in real-life situations, as well as a new paradigm to reveal unknown implicit aspects of information integration for emotional judgment.

## Introduction

Past studies have revealed some seemingly irrational aspects of the human mind in decision-making tasks. An example is the influence of task-irrelevant information such as the Simon effect (Simon and Craft, 1972). This influence is considered irrational because an ideally rational decision maker should not be affected by any task-irrelevant information. Another example can be found in the same–different task. In such a task, the reaction time for the same response were longer when the stimuli were different in task-irrelevant dimensions than when they were the same; thus, the task-irrelevant information could not be ignored completely (Egeth, 1966). Garner (1974) reported that either facilitation or interference would occur in response to non-emotional tasks (i.e., classification) depending on the nature of combined dimensions, and summarized the manner of information integration in visual spatial patterns and in auditory temporal patterns. The magnitude of the influence depends on the nature and the combinations of dimensions (Watanabe, 1988). A task-irrelevant influence can also be seen in emotional decision-making, such as visual attractiveness judgment, even when it is shown under our perception threshold (Murphy and Zajonc, 1993).

The specific question we address here is related to these findings: how can emotional values among different types of object be integrated spatially? In particular, would such “attractiveness leakage” occur even when the observer intends to ignore surrounding objects and to concentrate on a target object (Mier et al., 2011; Shimojo et al., 2011)? The significance of answering this question theoretically in relation to real-world applications should be obvious (think of advertisements in magazines or TV commercials, for instance). When one views an entire visual scene, perceptual organization/integration occurs mostly in a bottom up, task-irrelevant fashion (Kanizsa, 1979). It raises an intriguing possibility that the attractiveness of task-irrelevant visual stimuli, while concurrently presented with those that are task-relevant, may affect the attractiveness of the latter depending on the perceptual organization among them. Bearing in mind such motivations, we chose human faces and hairstyles as the stimuli in the current study. The face–hair pair is of utmost interest in this regard because one may expect a maximum degree of leakage owing to their tight perceptual organization.

The human has a well-developed ability to detect, recognize, and discriminate faces automatically, and to draw information from them. Needless to say, a face carries important social information. For instance, beauty is associated with goodness (Dion et al., 1972), earning potential (Elder, 1969), and advantage in mate choice (Thornhill et al., 1995). As for facial attractiveness, averageness, symmetry, and sexual dimorphism make faces more attractive (Langlois and Roggman, 1990; Grammer and Thornhill, 1994; Kowner, 1996; Perrett et al., 1998; Rhodes, 2006, for review). Holistic processing is important in facial attractiveness judgment (Abbas and Duchaine, 2008), and differences in eye movements during holistic and analytic processing of facial attractiveness has been noted (Schwarzer et al., 2005).

In modern perceptual studies about the face, during and after the 80 s in particular, stimuli tend to be prepared by cropping the face to eliminate hair, or by using computer-generated graphics that did not have hair. The influence from hair was considered a sort of artifact in the laboratory, and thus rarely examined. In reality, however, face is typically accompanied with hair. It is therefore rather natural to assume that the impression of one’s hair (i.e., hairstyle, hair color, etc.), or a lack of it, influences how the face looks. In fact, the hair plays an important role in some aspects of facial recognition in the real world, for example, in describing photos containing faces and in memory tasks (Davies et al., 1981). There is also evidence that one’s hair influences how one looks, e.g., in terms of physical attractiveness, health, and fertility (Swami et al., 2008), as well as personality (Graham and Jouhar, 1981). However, relatively little research has been conducted on hair attractiveness and its influence on the face, partly for the reason mentioned above.

In the current study, we investigated how the attractiveness of task-relevant and task-irrelevant objects (face/hair, or hair/face) is integrated in attractiveness evaluation. We also tracked eye movements during the evaluation task for an objective assessment of the participant’s overt attention. If our evaluation of facial or hair attractiveness is influenced by the perceptual misattribution of task-irrelevant facial and hair information, this phenomenon might be found for both male and female face and hair. However, past studies have suggested that the process of evaluating facial attractiveness differs when in evaluating male or female faces. Since both facial and hair makeup have been shown to manipulate appearance and attractiveness ratings of female models (Graham and Jouhar, 1981; Etcoff et al., 2011), we focused on the evaluation of female models in the current study.

## Experiments 1a,b

### Method

#### Participants

Thirty-one adults between the ages of 19 to 33 (*M* = 23.2 years, SD = 4.3 years, 14 females) participated in Experiment 1. Nineteen of the participants (*M* = 23.9 years, SD = 4.4 years, 9 females) participated in Experiment 1a, in which they viewed images of faces, hairs, and composites of faces and hairstyles to evaluate attractiveness, and 12 (*M* = 22.0 years, SD = 4.2 years, 5 females) participated in Experiment 1b, in which their eye movements during the sessions were recorded in addition to the evaluation task. All were naive about the purpose of the experiment, and had normal or corrected-to-normal vision. All of them were unfamiliar with the face and hair images used in the experiment. The Caltech Committee for the Protection of Human Subjects approved the experiment protocol, and informed consent was obtained from all the participants.

#### Materials and Stimuli

To simulate the diversity of faces in the real world, we included faces from multiple ethnicities and attractiveness levels in a stimulus set to use in Experiments 1a,b. Eight face images with four ethnicities (African, European, East Asian, and South Asian) and two attractiveness levels (attractive and less attractive) were selected from a pre-rated, larger set described in our past study (Park et al., 2010). All face images in the set were generated using FaceGen Modeller (Singular Inversions, Toronto, ON, Canada) and race categorization was based on that of the software. From the set of young female faces that consisted of 32 African faces, 36 East Asian faces, 30 European faces, and 38 South Asian faces, we selected the faces at the top 5% of attractiveness within each ethnic category as the attractive faces, and those at the bottom 5% within each ethnic category as unattractive faces. In addition, one face from the European category at the bottom 1% was added to this face set, because European faces at the bottom 5% were consistently evaluated as more attractive compared to the faces in other ethnicities. Sixteen images of hairstyles with two levels of length (long and short), two texture (straight and wave), and four colors (light blonde, darker blonde, light brown, and dark brown) were generated using the online software Hollywood Makeover (http://www.instyle.com/makeover) to include various colors and styles of hair. Each face image and each hair image were combined in the natural spatial alignment to make 144 face-and-hair composites. Experiments were written in Matlab using the Psychophysics Toolbox extensions (Brainard, 1997; Pelli, 1997; Kleiner et al., 2007).

#### Procedures

**FIGURE 1:**
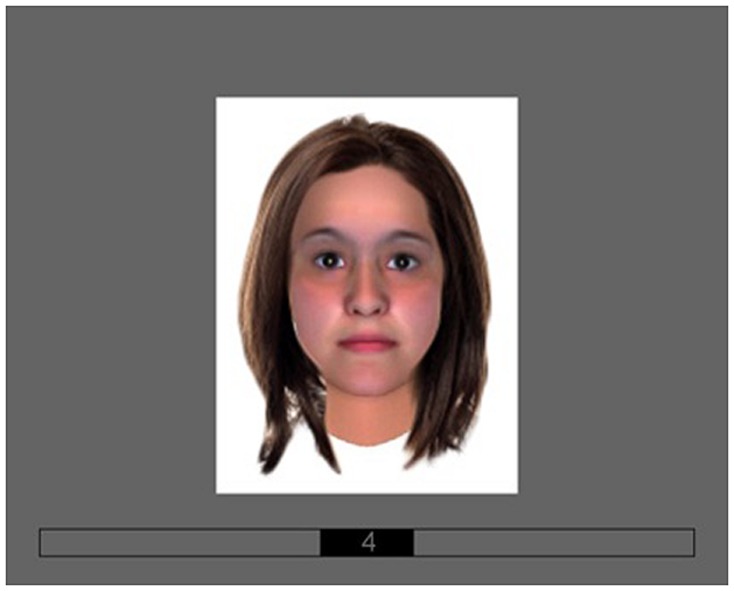
**Example of a composite stimulus presented on a CRT monitor for evaluation**. The hairstyle in this figure is a sample image, similar to (but different from) those used in the experiment. It was constructed from an image provided by stock images available at FreeDigitalPhotos.net.

There were two main sessions in which (i) face-only images (FO) and face–hair composites were randomly shown and (ii) hair-only images (HO) and face–hair composites were randomly shown. The participants were asked to evaluate the attractiveness of (i) face only or (ii) hair only on a 7-point scale (1: the least attractive, 4: neutral, 7: the most attractive), while ignoring task-irrelevant hair or face in the composite stimuli (for a sample of composite image trial, see **Figure 1**). In another control session (iii), in which only composites were shown, participants evaluated overall attractiveness. This third session was added to examine the possibility that the leakage effect in (i) or (ii) above may be due to the weighted average of face and hair. The images were presented on a 19-inch ViewSonic CRT screen at 1280 × 1024 pixel resolution with 60 Hz refresh rate. The order of sessions was randomized between participants, and the order of images within each session was also randomized. In all three sessions, a scale bar was presented at the bottom of the computer screen and participants rated the attractiveness using a mouse. A chin rest was set at a distance of 57 cm from the computer screen. In Experiment 1a, each stimulus had a size of 381 × 500 pixels, where face area was approximately 5.0^∘^ × 7.0^∘^ of visual angle. Each stimulus was presented on a larger scale in Experiment 1b for recording eye movements, where the size of the stimulus was 534 × 700 pixels with approximately 7.0^∘^ × 9.8^∘^ of face area. Eye movements during the sessions were recorded with a head-mounted, video-based eye tracker Eyelink-II (SR Research Ltd., Otawa, ON, Canada) at 250 Hz sampling rate, pupil-tracking mode. Nine-point calibration and validation in the settings of Eyelink-II was performed at the beginning of each session, and a drift correction was performed at the beginning of each trial.

#### Analyses

**FIGURE 2:**
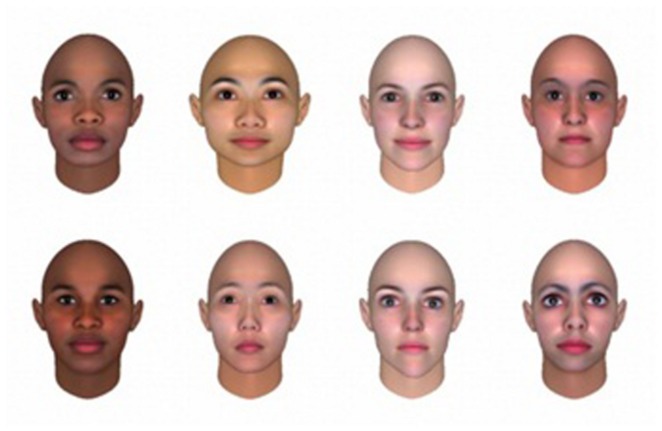
**A set of faces used in Experiments 1a,b**. In this figure, the set of faces only that were used in Experiments is shown. Face–hair composites were constructed by combining all of the faces with each of the 16 hairstyles.

In the analyses, data on the 16 hairstyles and 8 faces (a European face that was at the bottom 5% was excluded so that there would be an equal number of attractive faces and less attractive faces in the data set for analyses) shown in **Figure 2** were used. For the analyses of self-reported evaluation scores, the scores given by participants in Experiments 1a,b were pooled. Rating scores (*x*) were converted to *z*-scores (*z*) within each participant, using the mean (μ) and SD (σ) of the scores that each participant gave in all three sessions [*z* = (*x*–μ)/σ]. The mean scores were calculated for face attractiveness evaluation on FO, face attractiveness evaluation on face-and-hair composites (FC), hair attractiveness evaluation on HO, hair attractiveness evaluation on face-and-hair composites (HC), respectively, within each participant. The scores of FO and FC, as well as those of HO and HC, were compared using a dependent *t*-test to investigate if there was an influence from task-irrelevant hair in FC or from task-irrelevant face in HC. Eye movement was analyzed using Eyelink Data Viewer (SR Research Ltd.). To examine whether their gaze had been limited within the task-relevant area, the area of interests was defined by experimenter’s eyes, and the proportion of duration when their gaze was dwelling on the hair area over the duration when their gaze was dwelling in the area of the face-and-hair composite (dwell time ratio in hair area: DwR_H_) were calculated, and averaged over all the samples within each experimental condition within each participant. The difference in DwR_H_ between the conditions was then tested with a Friedman’s rank test.

### Results and Discussion

#### Attractiveness Ratings

**FIGURE 3:**
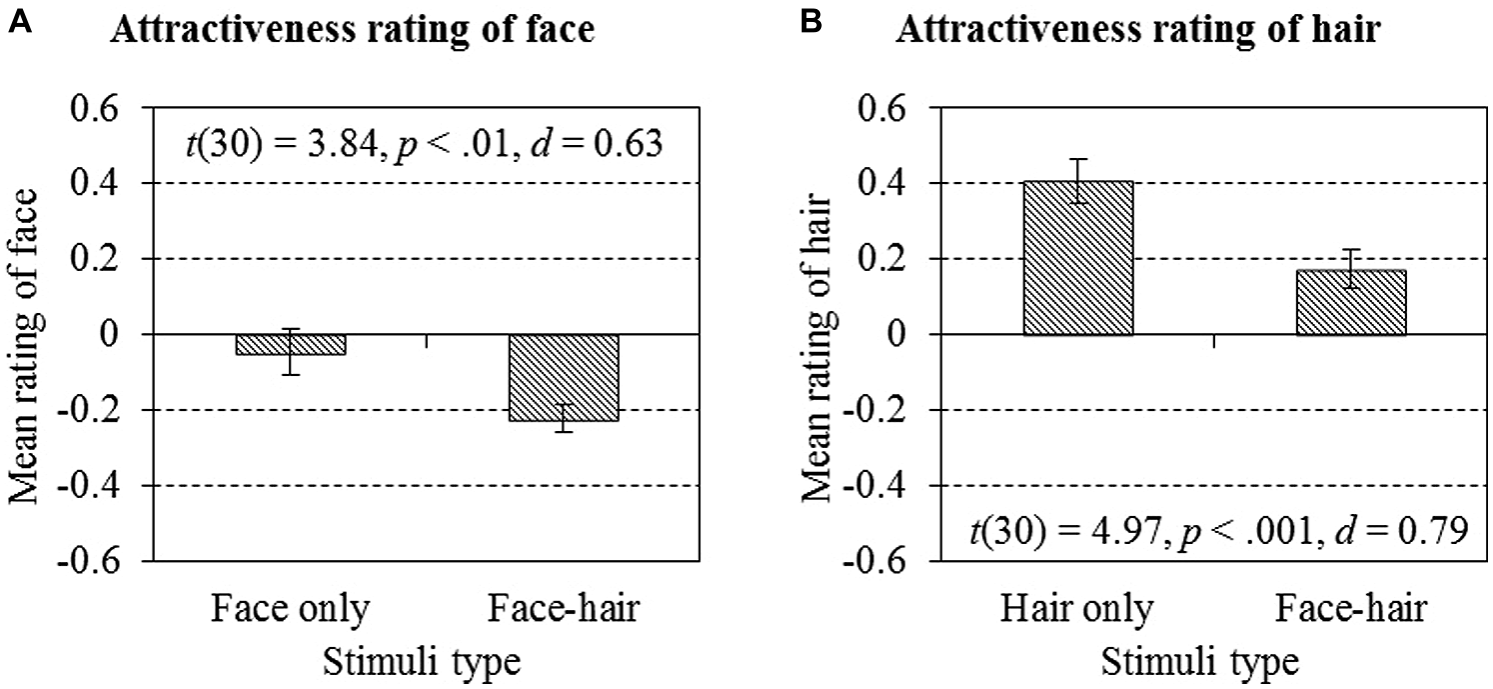
**Differences in attractiveness rating of faces when evaluating face-only stimuli and in evaluating face–hair composites **(A)**, and differences in attractiveness rating of hairstyles when evaluating hair-only stimuli and face–hair composites (B)**. In both cases, the ratings were significantly higher when evaluating target-only stimuli compared to that when evaluating face-and-hair composites [*t*(30) = 3.84, *p* < 0.01, *d* = 0.63 for face ratings and *t*(30) = 4.97, *p* < 0.001, *d* = 0.79 for hair ratings]. Error bars represent ±1 SEM.

Mean attractiveness ratings of faces were significantly higher when evaluating face-only stimuli [*M* = –0.049, SE = 0.61, 95% CI (–0.17, 0.076)] compared to that when evaluating face-and-hair composites [*M* = –0.22, SE = 0.036, 95% CI (–0.30, –0.15); *t*(30) = 3.84, *p* < 0.01, *d* = 0.63, 95% CI of the difference (0.081, 0.27)], as shown in **Figure 3A**. Similarly, mean attractiveness ratings of hair were significantly higher when evaluating hair-only stimuli [*M* = 0.41, SE = 0.057, 95% CI (0.29, 0.52)] compared to that when evaluating face-and-hair composites [*M* = 0.17, SE = 0.051, 95% CI (0.067, 0.28); *t*(30) = 4.97, *p* < 0.001, *d* = 0.79, 95% CI of the difference (0.14, 0.33); **Figure 3B**]. Thus, in both situations, the perceived attractiveness of the target (face or hair) was lower when evaluating face-and-hair composites compared to that when evaluating face-only or hairstyle-only stimuli. Because the faces presented in the FO condition and the FC condition, and the hairstyles presented in the HO condition and in the HC condition, were the same, the difference in attractiveness scores between the conditions indicate an influence on the evaluation of the target face or hair by the presence of task-irrelevant hair or face. The result that perceived attractiveness level decreased when face and hair were combined, for both the evaluations of face and hair, is rather paradoxical, since combining the face and hair is more realistic compared to face-only or hair-only stimuli. One possibility for this decrease might be due to the congruency between face and hair, since some combinations in our stimuli set (e.g., blonde hair with East Asian face) might be perceived as artificial.

It is known that facial attractiveness is evaluated differently depending on the evaluator’s gender. This suggests a possible gender difference in the influence from task-irrelevant face or hair on the evaluation of hair or face. To investigate this gender difference, a two-way repeated measures analysis of variance (ANOVA) was performed on the mean attractiveness scores of faces with presented stimuli type (FO or FC) as a within-participant factor and participants’ gender as a between-participant factor. The results indicated no interaction between evaluations of face with/without hairs and evaluator’s gender [*F*(1,29) = 0.792, *p* = 0.381, $\eta_{p}^{2}$ = 0.027]. Another ANOVA conducted on the mean attractiveness scores of hairstyle revealed no difference between evaluator’s gender in the influence from task-irrelevant face in evaluating hair [for the interaction, *F*(1,29) = 0.376, *p* = 0.545, $\eta_{p}^{2}$ = 0.013].

Results of the dependent *t*-tests indicated that the facial attractiveness evaluation in FO and FC, as well as the hair attractiveness evaluation in HO and HC, might be different from each other, but it is still unclear in what manner the influence occurs. A possible explanation is the misattribution of the attractiveness of task-irrelevant stimulus to the target stimulus. Thus, we investigated this prediction using a correlation analysis on the mean attractiveness score of each of the 8 faces in the FO condition and the average score of hairstyles that were presented with each of the eight faces in the HC condition, as an index of attractiveness leakage from face to hair. The results revealed a significant positive correlation [*r*(6) = 0.631, *p* < 0.05, one-tailed]. On the other hand, the index of attractiveness leakage from hair to face showed no significant correlation [*r*(14) = 0.275, *p* = 0.151, one-tailed]. The mean attractiveness evaluation given to each of the eight FO varied from –1.13 to 1.28, and a one-way repeated measures ANOVA revealed that the attractiveness level of FO significantly differed between the faces [*F*(4.13,123.9) = 27.3, *p* < 0.001, $\eta_{p}^{2}$ = 0.477]. Analogous to this, the means of the attractiveness scores given to the 16 hair-only stimuli varied from –0.49 to 1.25 and differed significantly between the hairstyles [with a Friedman’s rank test, χ^2^(15) = 112.3, *p* < 0.001], indicating that the manipulation of hair attractiveness was also successful. Although the variety of faces and hairstyles included in our stimuli set was rather small, and therefore, the interpretation of the findings should be limited to this stimulus set, the results nevertheless suggest the possible misattribution of facial attractiveness to the hair attractiveness evaluation. To summarize the findings, the attractiveness evaluation of hairstyles was influenced by that of task-irrelevant faces. Although the attractiveness evaluation of faces was influenced by the presence of hairs, it was unclear if the attractiveness of the hairstyle was a source of influence.

#### Eye Movement

**FIGURE 4:**
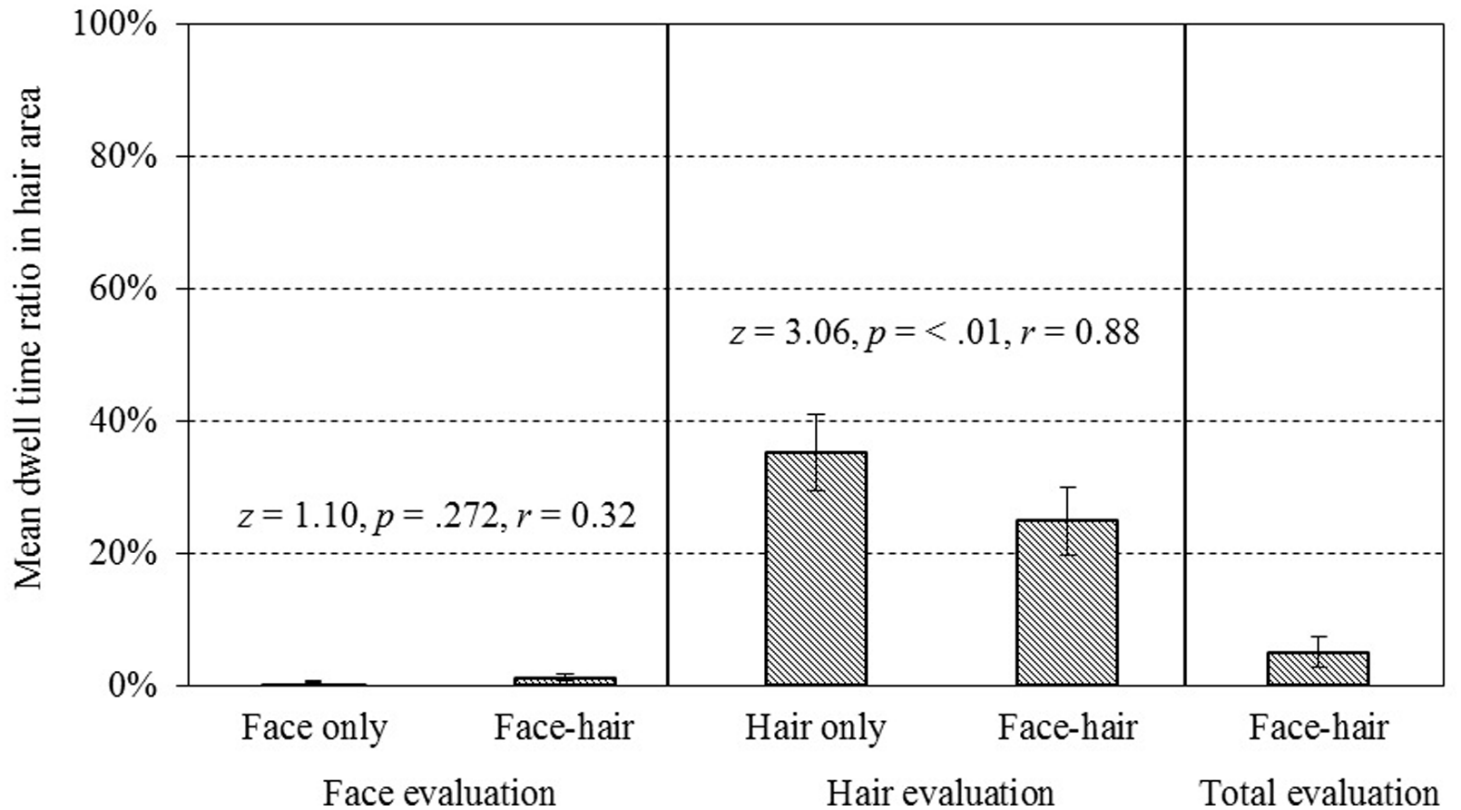
**The dwell time ratio of eye gaze within the hair area while performing each task (DwR_H_)**. A Friedman’s rank test showed a significant main effect of conditions [χ^2^(4) = 39.9, *p* < 0.001]. DwR_H_ in hair attractiveness evaluation for face–hair composites was significantly lower than that for hair-only stimuli (*z* = 3.06, *p* = < 0.01, *r* = 0.88 with Wilcoxon’s signed rank test), indicating that participants’ gaze was attracted to the face area when face–hair composites were presented. Error bars represent ± 1 SEM.

**FIGURE 5:**
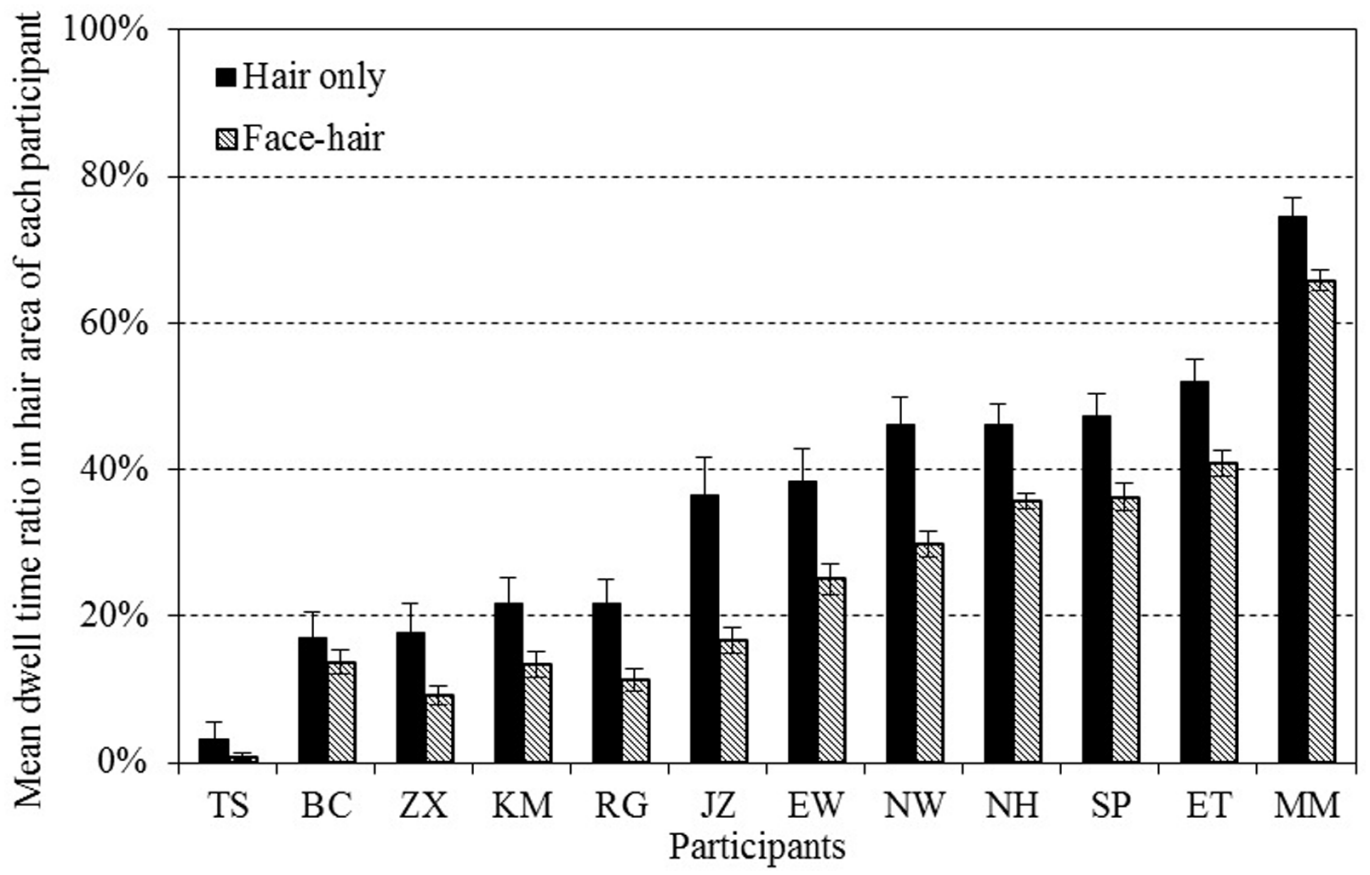
**The dwell time ratio of eye gaze within the hair area (DwR_H_) of each participant in the hair attractiveness evaluation task**. Although the ratio varied across participants, the DwR_H_ in the trials with face–hair composites was lower than the trials with hair-only composites in all of them. Error bars in the graph represent ± 1 SEM.

A Friedman’s rank test on DwR_H_ suggested a difference in dwelling time in the face/hair area between experimental conditions [χ^2^(4) = 39.9, *p* < 0.001; see **Figure 4**]. The conditions considered in the test were face attractiveness evaluation on FO, face attractiveness evaluation on face-and-hair composites (FC), hair attractiveness evaluation on HO, hair attractiveness evaluation on face-and-hair composites (HC), and overall attractiveness evaluation of face-and-hair composites (TC). Results of the Wilcoxon signed-rank test to compare DwR_H_ in HO and in HC revealed that DwR_H_ in HC [*Mdn* = 0.21, 95% CI (0.14, 0.36)] was significantly lower than that in HO [*Mdn* = 0.38, 95% CI (0.23, 0.48)], *z* = 3.06, *p* < 0.01, *r* = 0.88. This could be interpreted as the automatic drawing of participants’ gazes to the face if hair (the target) was presented with a face. In the face evaluation session, participants’ gaze was mainly in the face area regardless of whether there was hair, and there was no difference between DwR_H_ in FO [*Mdn* = 0.00, 95% CI (0.0005, 0.01)] and that in FC [*Mdn* = 0.01, 95% CI (0.001, 0.02)], *z* = 1.10, *p* = 0.27, *r* = 0.32. Thus, this effect was observed in only the hair attractiveness evaluation task. Although the sample size for the eye movements might be rather small, all the participants in Experiment 1b showed the same tendency in their gaze behavior in the hair attractiveness evaluation (**Figure 5**). There are two possible interpretations of this asymmetry. The first is the center-of-gravity in gaze behavior, which in effect would decrease the DwR_H_ in all cases, and the other is the salience of the face (relative to hair). It is not clear at this point which of these interpretations is more appropriate, since we cannot isolate these factors from each other in our choice of stimuli (frontal views of faces and hairstyles).

## Experiment 2

The results of Experiment 1 suggested a possible asymmetrical relationship between face and hair in the leakage of attractiveness from one to another. However, the variances of the baseline attractiveness of the faces and hairstyles were insufficient to allow discrimination between different predictions. Moreover, the findings in Experiment 1 indicated that the same hairstyles were perceived as less attractive when they were presented with a face compared to when they were presented in isolation. The same occurred in the attractiveness evaluation of faces. This might be due to incongruences within the face-and-hair pairs in Experiment 1. Since the stimuli set included face images of several ethnicities, matching between the ethnicities and hair color varied from unnatural to natural (e.g., blonde hairs were perceived as natural on European faces, but might be perceived as artificial on East Asian faces), which might have added noise to the results. Another potential cause of the noisy data was influence from stimuli repetition, since each face or hair was shown repeatedly (but combined with different hair or face) in a session. There also might be an artifact from the design, due to randomization of two types of stimuli (e.g., having face-only and face-with-hair) in a single session, which might have yielded some effect over trials, such as confusion over the task or a cognitive set in participants. To eliminate these factors in order to more sensitively detect attractiveness leakage effects, we conducted Experiment 2, in which only European faces were used. In addition, the attractiveness of both hair and face were exaggerated to maximize the leakage effect, and no repetition of face or hair were allowed in a session.

### Method

#### Participants

Thirty-two adults aged between 18 and 36 (*M* = 23.3, SD = 4.0, 9 females) were divided into two groups. One group performed a task set for investigating attractiveness leakage from face to hair and another group performed a set for the leakage from hair to face. The experiment protocol was approved by Caltech Committee for the Protection of Human Subjects, and informed consent was obtained from all the participants.

#### Materials and Stimuli

**FIGURE 6:**
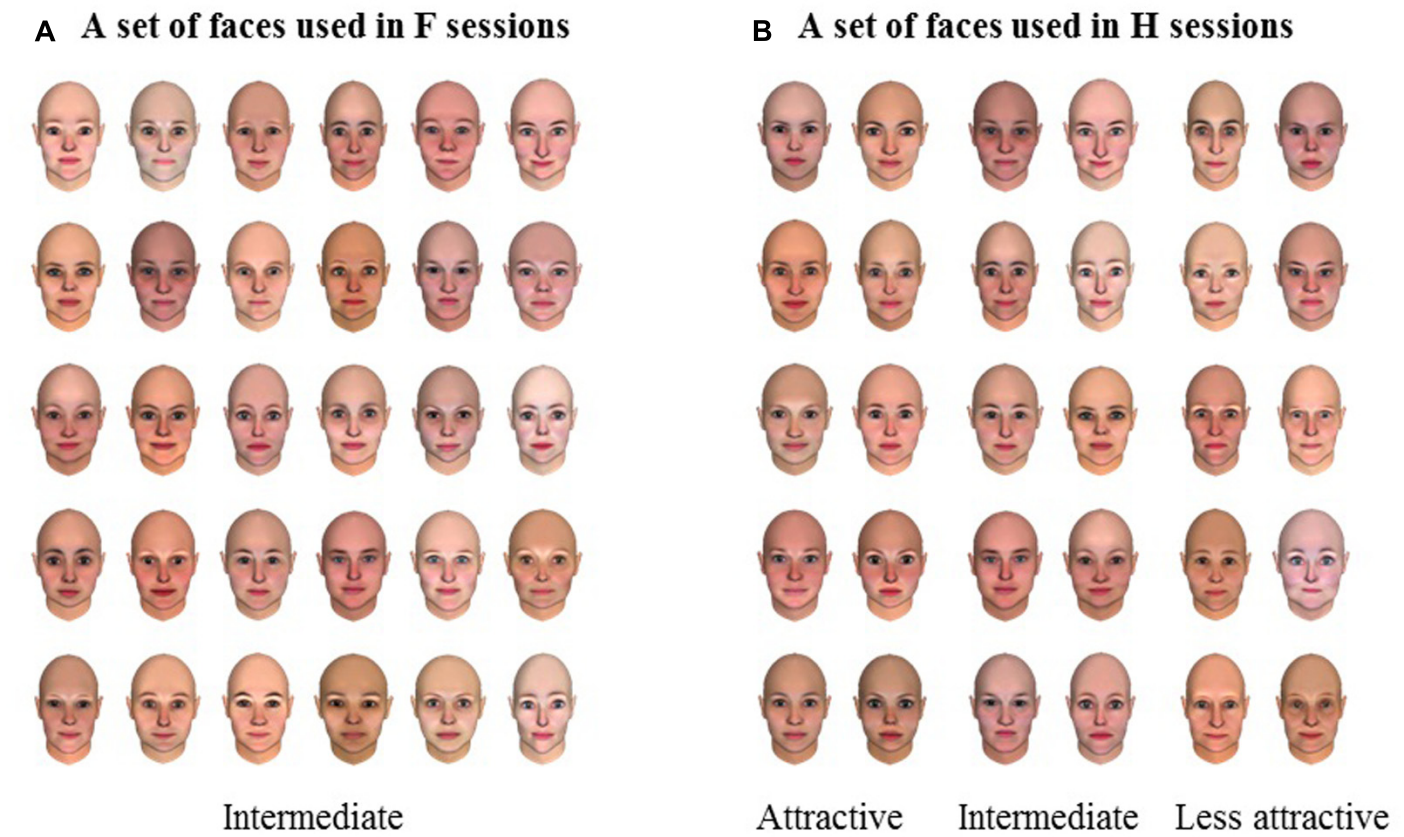
**Sets of faces used in F sessions (A) and H sessions (B) of Experiment 2**. In this figure the sets of faces only that were used in Experiment 2 are shown. The main task was face attractiveness rating in F sessions, while the main task was hair attractiveness rating in H sessions. The list of sessions is shown in **Figure 7**.

To create a stimulus set for investigating the leakage from face to hair, 30 hairstyles with intermediate level of attractiveness were selected from a pre-rated set of 128 hairs. Ten attractive, 10 intermediate, and 10 less attractive faces were selected from a pre-rated set of 140 European female faces generated using FaceGen Modeller. The hairs were divided into three groups of 10 hairs having an approximately similar level of mean attractiveness (according to the pre-rating given by a different set of participants) and proportion of characteristics such as color, shape, and length, between the groups. Then, each group of hairs was combined with each attractiveness level of face images to produce 10 composites of intermediate hair and attractive face, 10 of intermediate hairs and intermediate face, and 10 of intermediate hairs and less attractive faces. Similarly, 30 hair-and-face composites (i.e., three levels of hair attractiveness combined always with intermediate attractive faces) were prepared for investigating the attractiveness leakage from hair to face. These combinations were meant to maximize the potential leakage effect. **Figures 6A,B** shows the sets of faces only that were used in experiments.

#### Procedures

**FIGURE 7:**
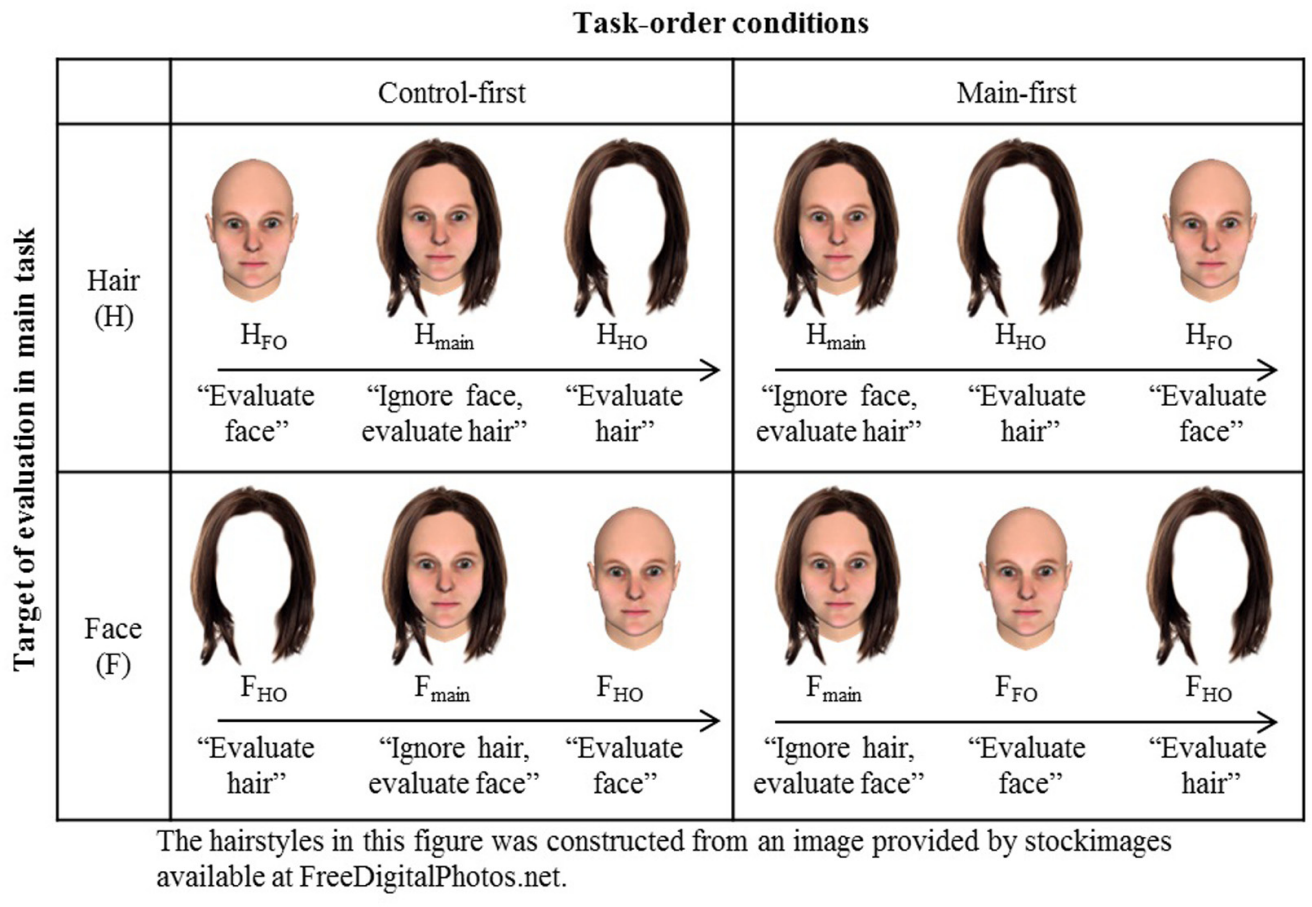
**List of conditions in Experiment 2**. The abbreviations “H” or “F” were assigned to each session depending on the target (hair: H or face: F) to be evaluated in the main task, where task-irrelevant face or hair should be ignored. Participants were randomly assigned to two types of task order. Note that, in this figure, hairstyle is a sample image, similar to (but different from) the stimuli used in the experiment.

The task set for the attractiveness leakage from face to hair consisted of three sessions. In the main session (H_main_), face-and-hair composites were shown in pseudo-random order in which the order of the composites were pre-determined to allocate the attractiveness levels evenly throughout the session (“H” indicates that hair attractiveness rating was the main task, while “F” indicates that face attractiveness rating was the main task, throughout this paper. See **Figure 7** for the list of conditions.) Two patterns of pre-determined pseudo random order were generated, and randomly assigned to participants. Participants were asked to ignore the face and evaluate the attractiveness of only hair on a 7-point scale (1: the least attractive, 4: neutral, and 7: the most attractive). In the two control sessions (hair-only session: H_HO_, and face-only session: H_FO_, respectively), hair or face images that were shown in the main session were presented alone without face or hair, and participants were asked to rate the attractiveness of them again on the 7-point scale. For all three sessions, each image was viewed for 0.5 s before the rating. This procedure was meant to give the participants an idea of the possible range of attractiveness. The control session H_HO_ was conducted to secure the same attractiveness levels of hairs in the three groups, and the other control session H_FO_ was conducted to secure the attractiveness threshold in the “to-be-ignored” faces. As shown in **Figure 7**, half of the participants were assigned to the “main-first” group, where the task order was H_main_, H_HO_, and then H_FO_. Another half of the participants were assigned to the “control-first” group, where the task order was H_FO_, H_main_, and then H_HO_. These two orders were set to examine and to balance order effects due to task order. Likewise, the task set for the attractiveness leakage from hair to face consisted of a main session (F_main_) where participants saw composite images and rated the attractiveness of only the face while ignoring the hair, and two control tasks F_FO_ and F_HO_ where they rated the attractiveness of face-only or HO. As before, there were two session orders (“main-first” and “control-first”).

Eye movement was recorded using the same equipment and settings as in Experiment 1. Since the recording was not successful for one participant in the “main-first” group of the leakage from the hair to face task set, eye movement analysis was based on data from 31 participants.

A *post hoc* questionnaire was completed after the experiment to check if participants followed the instruction to ignore task-irrelevant face or hair and also to check if they noticed any influence from the task-irrelevant stimuli.

#### Analyses

All the rating scores were standardized as described in Experiment 1. To investigate the influence of the attractiveness level of the task-irrelevant hairs (or that of the task-irrelevant faces) on the attractiveness evaluation of faces (or that of hairs), two-way ANOVAs were performed on the attractiveness ratings in the main and baseline sessions, with the stimuli type and the attractiveness level of task-irrelevant face or hair as repeated measure factors. As in Experiment 1, we employed *post hoc* tests to examine gender differences in the attractiveness self-reports (ratings of attractiveness). We performed two-way ANOVAs on the ratings (for H_main_ and F_main_) with attractiveness of the to-be-ignored face or hair as the within-participants factor and gender as the between-participants factor.

When analyzing eye movement, the data from the first trial of each session was eliminated due to a longer response time compared to in other trials. Then, dwell-time ratio in task-irrelevant face (or hair) area was calculated in a way similar to that in Experiment 1. In addition, saccade amplitude during the sessions was calculated as another index of eye movement in Experiment 2 to examine if the holistic/analytical processing of information is related to the attractiveness leakage phenomenon.

### Results and Discussion

#### Attractiveness Ratings

##### Attractiveness leakage from face to hair

**FIGURE 8:**
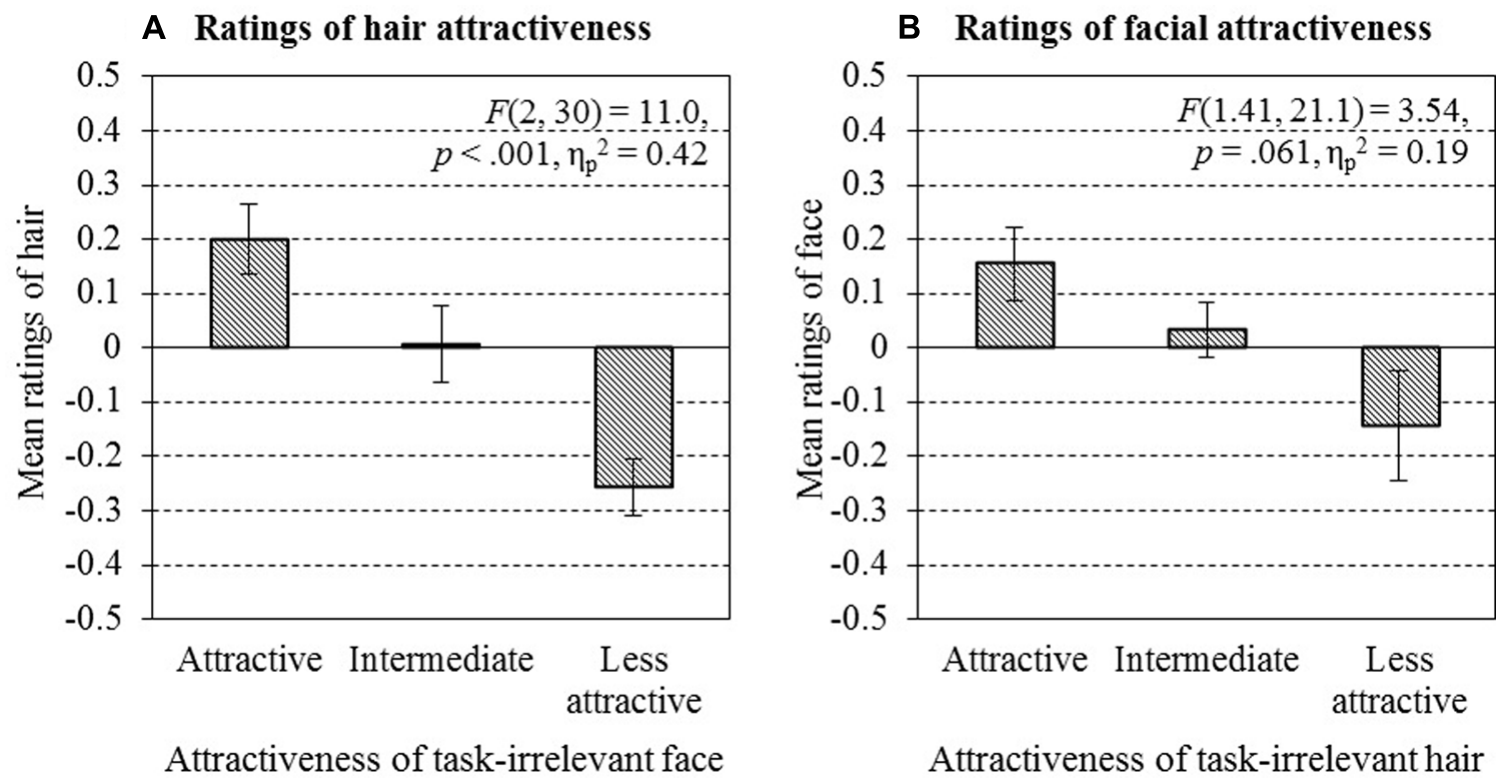
**Attractiveness ratings of hair presented with task-irrelevant face (A) and those of face presented with task-irrelevant hair (B)**. Error bars represent ±SEM. ANOVAs showed a significant influence of task-irrelevant face on hair [*F*(2,30) = 11.0, *p* < 0.001, $\eta_{p}^{2}$ = 0.42] and a marginal influence of task-irrelevant hair on face [*F*(1.41,21.1) = 3.54, *p* = 0.061, **$\eta_{p}^{2}$** = 0.19], using degrees of freedom corrected with Greenhouse–Geisser estimates of the sphericity.

A two-way ANOVA on mean attractiveness ratings in H_main_ and in H_HO_ was performed with attractiveness levels of task-irrelevant face (attractive, intermediate, or less attractive) shown with the hair as a within-participant factor. There was a significant main effect of task-irrelevant facial attractiveness level [*F*(2,30) = 5.57, *p* < 0.01, $\eta_{p}^{2}$ = 0.27], indicating the presence of attractiveness leakage from face to hair, a significant main effect of layout condition [H_main_ or H_HO_: *F*(1,15) = 7.21, *p* < 0.05, $\eta_{p}^{2}$ = 0.33], and a significant interaction between layout condition and facial attractiveness level [*F*(2,30) = 8.91, *p* < 0.01, $\eta_{p}^{2}$ = 0.37]. The main effect of layout condition showed that the ratings in H_main_ [*M* = –0.017, 95% CI (–0.076, 0.042)] were significantly lower than those in H_HO_ [*M* = 0.12, 95% CI (0.053, 0.19); *F*(1,15) = 11.6, *p* < 0.001, $\eta_{p}^{2}$ = 0.44, 95% CI of the difference (–0.25, –0.028)]. To interpret the interaction, *post hoc* repeated ANOVAs were performed within H_main_ and within H_HO_, respectively. In H_main_, the main effect of task-irrelevant facial attractiveness on hair attractiveness rating was significant [*F*(2,30) = 11.0, *p* < 0.001, $\eta_{p}^{2}$ = 0.42; see **Figure 8A**]. A posteriori Bonferroni analysis revealed a significantly lower hair attractiveness score in trials with less attractive faces [*M* = –0.26, SEM = 0.052, 95% CI (–0.37, –0.15)] compared to in trials with attractive faces [*M* = 0.20, SEM = 0.065, 95% CI (0.062, 0.34); *p* < 0.001, 95% CI of the difference (–0.68, –0.24)], and compared to in trials with intermediate faces [*M* = 0.006, SEM = 0.070, 95% CI (–0.14, 0.16); *p* < 0.01, 95% CI for the difference (–0.42, –0.11)]. However, there was no significant difference between trials with attractive faces and those with intermediate faces [*p* = 0.106, 95% CI for the difference (–0.046, 0.43)]. The lack of a significant main effect of ratings in H_HO_ [*F*(2,30) = 0.11, *p* = 0.896] indicates no effect from hair attractiveness itself, as expected, because we controlled the attractiveness level of all hair stimuli to be moderate. Thus, we can conclude, to a significant extent, the difference in hair ratings in H_main_ was due to task-irrelevant, to-be-ignored face attractiveness. This result is consistent with the attractiveness leakage phenomenon observed in Experiment 1.

An ANOVA investigating gender differences revealed no significant interaction between participants’ gender and attractiveness of the to-be-ignored face [*F*(2,28) = 0.98, *p* = 0.39, $\eta_{p}^{2}$ = 0.065].

Further, to double-check the leakage phenomena from face to hair, we performed a regression analysis with the attractiveness rating on target hair in H_main_ as the dependent variable and the other ratings (attractiveness ratings for hair in H_HO_ and face in H_FO_) as independent variables. Results indicated that attractiveness ratings for both independent variables made a significant positive contribution to attractiveness ratings in H_main_. Standardized coefficients were β = 0.59 for H_HO_ (*p* < 0.001) and β = 0.14 for H_FO_ (*p* < 0.001). The adjusted *R*^2^ value for the model was 0.35. Thus, although the variance is rather small and interpretations should be made carefully, this result may support the results of the ANOVAs that were performed on mean attractiveness ratings in H_main_ and in H_HO_.

##### Attractiveness leakage from hair to face

An analogous two-way repeated measure of ANOVA on mean ratings in F_main_ and those in F_FO_ were conducted. The results showed a significant main effect of hair attractiveness [*F*(2,30) = 3.77, *p* < 0.05, $\eta_{p}^{2}$ = 0.20), indicating a leakage from hair to face. There was no significant main effect of the layout condition [*F*(1,15) = 1.34, *p* = 0.26, $\eta_{p}^{2}$ = 0.082). *Post hoc* repeated-measures ANOVAs were performed on the ratings in F_main_ and on those in F_FO_ respectively. The main effect of the task-irrelevant hair attractiveness was only marginally significant in F_main_ [*F*(1.41,21.1) = 3.54, *p* = 0.061, $\eta_{p}^{2}$ = 0.19; degrees of freedom were corrected using Greenhouse–Geisser estimates of sphericity; see **Figure 8B**], while it was not significant in F_FO_ [*F*(2,30) = 2.26, *p* = 0.122, $\eta_{p}^{2}$ = 0.13].

**FIGURE 9:**
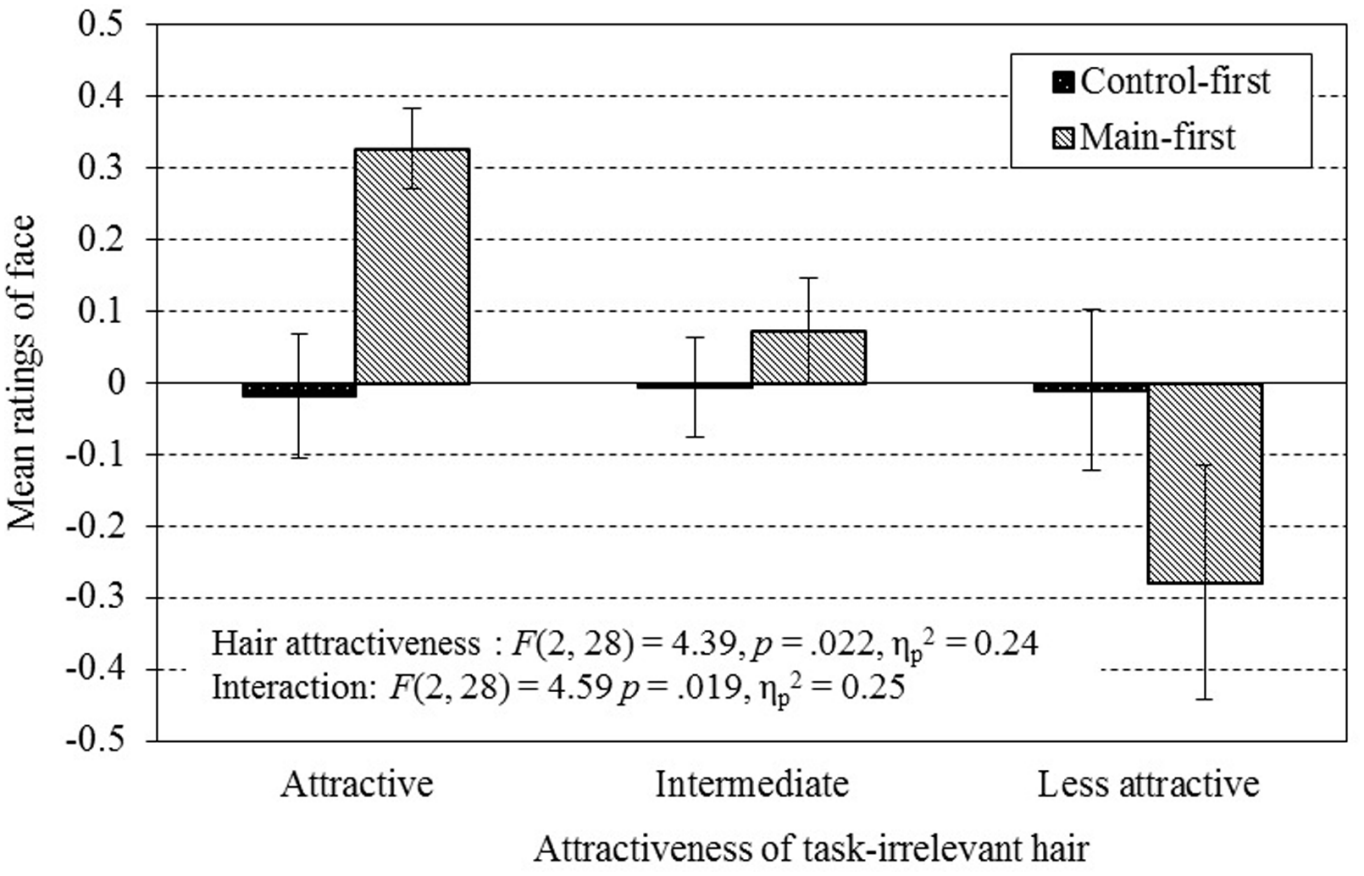
**Attractiveness ratings of face presented with task-irrelevant hair in control-first and in main-first task order**. There was a significant interaction between task-order and task-irrelevant hair attractiveness [*F*(2,28) = 4.59, *p* = 0.019, $\eta_{p}^{2}$ = 0.25), suggesting a possible difference in the influence from hair to face in attractiveness evaluation depending on the task order. Error bars represent ±SEM.

To interpret the marginal significance in the main effect of task-irrelevant hair attractiveness in F_main_, we investigated the possible influence of task order on the attractiveness leakage from hair to face by conducting a two-way ANOVA on the ratings in F_main_ with task-order as the between-participant factor and task-irrelevant hair attractiveness as the within-participant factor. The result demonstrated a significant interaction of task-order and task-irrelevant hair attractiveness [*F*(2,28) = 4.59, *p* < 0.05, $\eta_{p}^{2}$ = 0.25], as well as a significant main effect of task-irrelevant hair attractiveness [*F*(2,28) = 4.59, *p* < 0.05, $\eta_{p}^{2}$ = 0.25]. Subsequent one-way ANOVAs conducted on face ratings on the attractiveness level of task-irrelevant hair within the “main-first” and “control-first” group respectively showed that the main effect of task-irrelevant hair attractiveness was significant in the “main-first” group [*F*(2,14) = 7.05, *p* < 0.01, $\eta_{p}^{2}$ = 0.50] but not in the “control-first” group [*F*(2,14) = 0.005, *p* = 1.00, $\eta_{p}^{2}$ = 0.001; see **Figure 9**]. These results suggest that the significant main effect of task-irrelevant hair attractiveness in the first ANOVA is mainly due to the “main-first” group, which is free from a sequential effect across sessions. Although the sample sizes for the *post hoc* ANOVAs were rather small, the results nevertheless suggest that the influence from task-irrelevant hair on the attractiveness evaluation of the face differs depending on whether participants were familiar with the hairstyles before starting the main session.

There was no significant difference between male and female participants in the influence of task-irrelevant hair attractiveness on the attractiveness evaluation of faces. That is, there was no significant interaction between participant gender and hair attractiveness: [*F*(1.44,21.6) = 1.84, *p* = 0.19, $\eta_{p}^{2}$ = 0.11, degrees of freedom corrected using Greenhouse–Geisser estimates of sphericity].

We double-checked the findings using multiple regression analyses of the attractiveness ratings in F_main_ and F_HO_. Because the ANOVA results suggested that attractiveness leakage might differ depending on task order, we analyzed ratings from the control-first and main-first groups, separately. The standard coefficients in the regression models were as follows: main-first task, β = 0.34 for F_FO_ (*p* < 0.001) and β = 0.20 for F_HO_ (*p* < 0.01); control-first condition, β = 0.35 for F_FO_ (*p* < 0.001) and β = –0.012 for F_HO_ (*p* = 0.84). Adjusted *R*^2^ values for the models were 0.14 (control-first) and 0.17 (main-first). The difference between the models can be interpreted as the effect of task-order on how faces are evaluated (for attractiveness) in the presence of task-irrelevant hair. This suggests changes in the mechanisms of facial attractiveness perception between the experimental conditions (control-first and main-first). Although this interpretation should be made with caution (for reasons described in the previous section), the results may support the findings from ANOVA performed on the ratings in F_main_.

#### Eye Movement

One-way ANOVAs with the attractiveness level of task-irrelevant hair as a within-subject factor were conducted on the mean dwell-time ratio in the hair area (DwR_H_) as well as on the mean saccade amplitude during F_main_. Likewise, a one-way ANOVA was conducted on saccade amplitude during F_main_. Regarding dwell-time ratio in the face area (DwR_F_) in F_main_, Friedman’s rank test was performed because the scores were not normally distributed.

##### Eye movements with the attractiveness leakage from face to hair

The results showed a significant main effect of task-irrelevant face attractiveness in saccade amplitude during H_main_ [*F*(2,28) = 4.11, *p* < 0.05, $\eta_{p}^{2}$ = 0.23]. A posteriori Bonferroni analysis revealed significantly smaller saccade amplitudes in trials with attractive faces [*M* = 3.31, SEM = 0.15, 95% CI (2.99, 3.63)] than in trials with intermediate faces [*M* = 3.49, SEM = 0.15, 95% CI (3.17, 3.81); *p* < 0.05, 95% CI of the difference (–0.34, –0.029)], whereas those in trials with less attractive faces [*M* = 3.41, SEM = 0.15, 95% CI (3.09, 3.73)] were not significantly different from either those in trials with attractive faces [*p* = 0.468, 95% CI of the difference (–0.29, 0.082)] or intermediate faces [*p* = 0.728, 95% CI of the difference (–0.099, 0.26)]. This could be interpreted in two possible ways. First, the attractive faces might strongly attract our eye gaze automatically, and thereby lead to smaller saccade amplitude. Second, the presence of an attractive face might have led to holistic processing, which in turn led to longer dwelling time on the nose area in the face, as indicated in the literature (Schwarzer et al., 2005). However, the difference between the mean angles are rather small (e.g., 0.18^∘^) and thus might not constitute a meaningful difference. Also, interpretations should be made carefully as the sample size was rather small. No significant effect was observed in DwR_H_ [*F*(2,28) = 0.25, *p* = 0.781, $\eta_{p}^{2}$ = 0.018].

##### Eye movements with attractiveness leakage from hair to face

There was no significant main effect of the attractiveness level of task-irrelevant hair in both saccade amplitude and in DwR_F_ [for saccade amplitude, *F*(1.44,21.7) = 1.26, *p* = 0.30, $\eta_{p}^{2}$ = 0.077, using corrected degrees of freedom estimated with Greenhouse–Geisser estimates for sphericity; for DwR_F_, χ^2^(2) = 0.32, *p* = 0.85].

## General Discussion

We examined if the attractiveness of a hairstyle (face) is implicitly affected by a face (hairstyle) in two experiments. Results from Experiment 1 provided evidence for the “attractiveness leakage” from face to hair, but not from hair to face, when examining the correlation coefficient as the leakage index. In Experiment 2, we adjusted the attractiveness levels of the faces and hairstyles used to maximize the leakage effect, and manipulated the session order to address a sequential order effect. The results showed significant bidirectional attractiveness leakage between face and hair in the “main-first” session order, and a unidirectional leakage from face to hair in the “control-first” session order, explaining the marginally significant result in Experiment 1 (i.e., no significant leakage from hair to face). In short, the attractiveness leakage is bidirectional between face and hair, as long as there is no prior experience with the same stimuli. Our findings are consistent with other findings from different stimuli/tasks (Egeth, 1966; Simon and Craft, 1972; Garner, 1974; Watanabe, 1988), in that (i) attractiveness evaluation was influenced by task-irrelevant visual information, and (ii) the influence was to some extent asymmetric in its direction. The asymmetry could probably be explained by the ability of our visual system that is tuned automatically to faces, but not so much to hairstyles.

Eye movement patterns in Experiment 1 were partly consistent with the asymmetric pattern of the attractiveness leakage. DwR_H_ (dwell time ratio in hair area) in the hair evaluation task was lower in the trials where hair was shown with face, while DwR_H_ in the face task was not influenced by the presence of hair. This is consistent with our hypothesis that the asymmetry in the leakage effect is due to the automaticity/priority of face processing in our visual system, assuming that overt attention shifts typically reflect covert attention shifts. Another way to interpret this finding is to take into account the visual information processing strategy. It is known that holistic processing plays an important role in facial attractiveness judgment (Abbas and Duchaine, 2008). As for the relationship between eye movement and visual processing, holistic processors tend to look more at the eye and nose area (Schwarzer et al., 2005). More interfeatural saccades are observed in the configural condition than in featural condition, and participants fixated at the center of face tended to perceive it in a holistic way in the condition cued by an intact face, compared to configural, or featural conditions that were cued by blurred or scrambled face (Bombari et al., 2009). Thus, the differences in DwR_H_ in Experiment 1 could also be interpreted in the processing strategy framework. However, the influence of the attractiveness level of the task-irrelevant face or hair on gaze behavior during the evaluation of the attractiveness of the task-relevant target remains unclear. Also, there is a possibility that the usage of a head-mounted eye tracker in our experiment might have influenced participants’ eye gaze behavior during the tasks. Thus, further research is required to determine the direction of causality.

A sequential effect observed in the face evaluation task in Experiment 2 might be interpreted along the same line. The control task in which participants evaluated the hair attractiveness of hair-only stimuli might prime a feature-based perceptual strategy (as in Bombari et al., 2009), thus interfering with a holistic perception in the subsequent main task with the face–hair composites. Another possible interpretation is the predictability of the range of hair attractiveness. A “control-first” task order might have enabled participants to learn the range of task-irrelevant hair attractiveness before proceeding to the main task, and such a cognitive set might have limited the implicit influence from the task-irrelevant stimulus in the subsequent session (F_main_).

In the context of affective states of human emotion, Schwarz and Clore (1983) reported that participants’ evaluation on general well-being was influenced by the weather of the day only when participants were not primed by the interviewer about the weather. A similar tendency was found in the mere exposure effect. Through a meta-analysis of research on Zajonc’s (1968) mere exposure effect, Bornstein (1989) revealed that a degree of implicitness/explicitness of the presented stimuli affected the effect size of the mere exposure effect. The more implicitly the stimulus was presented, the more influence it had. Jacoby and Whitehouse (1989) explained this phenomenon from the perspective of perceptual fluency, assuming that perceptual fluency underlies the mere exposure effect, and proposed that participants misattributed perceptual fluency to liking in a subliminal condition while engaging in a correction process in a supraliminal condition (Bornstein and D’Agostino, 1992). In terms of the current findings, it has been reported that perceptual fluency is involved in aesthetic evaluations (Reber et al., 2004). Thus, a sequential effect we observed might be explained due to either a predictability of the range of hair attractiveness or a priming to hair attractiveness.

In the *post hoc* questionnaire in Experiment 2, most participant reported that they noticed some influence from task-irrelevant stimuli (*M* = 3.95 and SD = 0.89 for ratings on a 5-point scale ranging from 1: “did not notice any influence” to 5: “noticed influence”), even though they tried to follow the instruction to ignore it (*M* = 4.68 and SD = 0.59 for ratings on a 5-point scale ranging from 1: “did not follow instruction” to 5: “followed instruction”). There were no noticeable differences in their answers according to the target stimuli (face or hair) or session order. This suggests that, in face attractiveness evaluation in the “control-first” task order, the participants noticed an influence from task-irrelevant hair and were able to suppress it. However, in the other conditions (face attractiveness in the “main-first” task-order condition and hair attractiveness in both of the task-order conditions), they could not suppress the influence from task-irrelevant stimuli.

The leakage effects we observed should still be considered partly “implicit” in the following ways: (1) affected by task-irrelevant stimuli, (2) participants followed instruction to ignore the task-irrelevant stimuli according to their eye movement behavior, and (3) even when they were aware of the influence (from the other part), they could not entirely cancel the effect through effort. However, most of the participants were aware of the influences from the other part of face, and in the particular condition/sequential order they could suppress the influence. Thus, we could conclude that both the explicit and implicit processes contributed to the attractiveness rating.

As an application to the real world, a misattribution of information to irrelevant objects has been researched in relation to advertisement. Especially, it is widely known that physically attractive models in advertisements influence consumer’s perception toward the advertisement itself and the advertised product (Baker and Churchill, 1977). Here, we showed that such a misattribution could be observed within a person, between facial and hair stimuli, indicating that such leakage could occur even at perceptual, rather than cognitive or contextual levels. Facial attractiveness is an important impression factor for women applying facial makeup, and past studies have revealed how facial makeup could change the perceived impression of a face (Graham and Jouhar, 1981). Our findings suggest that how one’s hair looks might influence how one’s face looks, and how one’s face looks might influence how one’s hair looks. Further, although we focused on the possible misattribution of attractiveness between face and hair, our findings may open up questions about attractiveness integration in general. Thus, our findings may have relevance in several fields including cognitive psychology, neuroscience, and behavioral economics (e.g., in marketing and consumer research). Also, as the faces used in our experiment were computer-generated realistic images, the possible differences between the perception of these faces and that of photos of real faces should be addressed in future research.

In summary, the evaluation of hair attractiveness was influenced by task-irrelevant face attractiveness regardless of the session order, whereas the evaluation of face attractiveness was influenced by task-irrelevant hair only when participants performed the task without influences from the prior task (of rating the baseline attractiveness of hairstyles). In other words, the leakage from hair to face occurs only in situations where the sequential effect is eliminated, i.e., situations that are more natural and consistent with the typical real-world context. Gaze behavior was consistent with the results and the interpretation. The asymmetry in the attractiveness leakage effect from face to hair and that from hair to face possibly indicates an asymmetry in perceptual and attentional processing between face and hair. Finally, combining the results from the *post hoc* questionnaire, eye movements, and behavioral data, both the implicit and explicit processes seem to contribute to the leakage effect.

The findings revealed another notable case of influence from task irrelevant stimuli, shedding new light on the implicit-explicit interplay in attractiveness judgment, especially in the face–hair stimuli. As such, it provides a new paradigm to explore the intricate relationship between perception and aesthetic decision under various contexts.

In the current study, we investigated behavioral and perceptual aspects of attractiveness leakage related to a task-irrelevant object by using face and hair as stimuli. We suggest that future fMRI or EEG studies may reveal the neural mechanisms underlying such integration processes in the assessment of visual attractiveness. Data from neuroimaging could complement those from eye movement research to provide a better understanding about holistic processing. Since the middle fusiform gyrus, as well as the inferior occipital gyrus, have been reported to support a holistic representation of faces (Schiltz and Rossion, 2006), these brain areas would likely be involved in the leakage phenomenon. Another possible account is the misattribution of emotion on a false target. In this case, emotion displayed on the unattended face would be automatically misattributed to the evaluation of the target stimuli. In fact, some research findings revealed linear neural responses in the reward centers in the brain such as the orbitofrontal cortex (O’Doherty et al., 2003), nucleus accumbens (Aharon et al., 2001; Kim et al., 2007), and ventral occipital region (Chatterjee et al., 2009) to attractive faces, even when people were performing an unrelated task, and non-linear responses in the amygdala, with the greatest responses to both the most attractive and least attractive faces (Winston et al., 2007). This possibility seems consistent with participants’ subjective experience that they could not cancel out the influence from unattended objects even when they were aware of the influence. Thus, understanding of the underlying mechanisms would be deepened by taking the effective behavioral paradigm described in the present study in the MRI scanner.

## Conflict of Interest Statement

This study was partly funded by Kao Corporation, where CS is employed as a researcher. The authors declare that the research was conducted in the absence of any commercial or financial relationships that could be construed as a potential conflict of interest.
